# Toward Two-Dimensional MoS_2_ Electrets with Modulated Fluorescence

**DOI:** 10.1021/acs.nanolett.6c03760

**Published:** 2026-10-16

**Authors:** Eugenio Lunedei, Andrea Liscio, Francesco Borgatti, Edoardo Chini, Pasquale D’Angelo, Fiorenza Esposito, Niccolò Borghi, Matteo Mannini, Denis Gentili, Luca Seravalli, Massimiliano Cavallini

**Affiliations:** † 9327 Istituto per lo Studio dei Materiali Nanostrutturati (ISMN) - Consiglio Nazionale delle Ricerche (CNR) Via P. Gobetti 101, 40129 Bologna, Italy; ‡ Istituto per la Microelettronica e Microsistemi (IMM) Consiglio Nazionale delle Ricerche (CNR), Rome unit, Via del fosso del cavaliere 100, 00133, Roma, Italy; § Istituto dei Materiali per l’Elettronica ed il Magnetismo (IMEM) - Consiglio Nazionale delle Ricerche (CNR) Parco Area delle Scienze, 37/A, 43124 Parma, Italy; ∥ Department of Mathematical, Physical and Computer Sciences, 9370 University of Parma, Parco, Area delle Scienze 7/a, 43124 Parma, Italy; ⊥ Dipartimento di Chimica “ U. Schiff”, Università di Firenze, Via della Lastruccia 3-13, 50019 Sesto Fiorentino, Italy

**Keywords:** MoS_2_ monolayer, Electret, Defect-Engineering, Charge trapping, Excitons and trions, Time resolved photoluminescence, Patterning

## Abstract

We show how spatially controlled sulfur vacancy engineering enables monolayer MoS_2_ to act as an electret, storing quasi-permanent electrostatic charge within a single atomic layer and modulating its emission properties. Sulfur vacancies are created by stamp-assisted, electrode-free electrochemical nanolithography, yielding tunable defect densities from 10^10^ to 10^13^ cm^–2^. The vacancy domains act as electron traps, resulting in surface charge density up to 1 μC cm^–2^, with charge retention exceeding 300 days under ambient conditions. Kelvin probe and electric force microscopies reveal electrostatic patterns replicating the lithographic motif. These domains also define a colocalized exciton-quenching regions in fluorescence microimages. They modulate the exciton fluorescence lifetime recorded at room temperature from tens of nanoseconds to a few hundred picoseconds by enhanced nonradiative exciton deactivation and induce a different balancing between excitons and trion emission via free electron draining from the untreated zones of the crystal.

## Graphical abstract

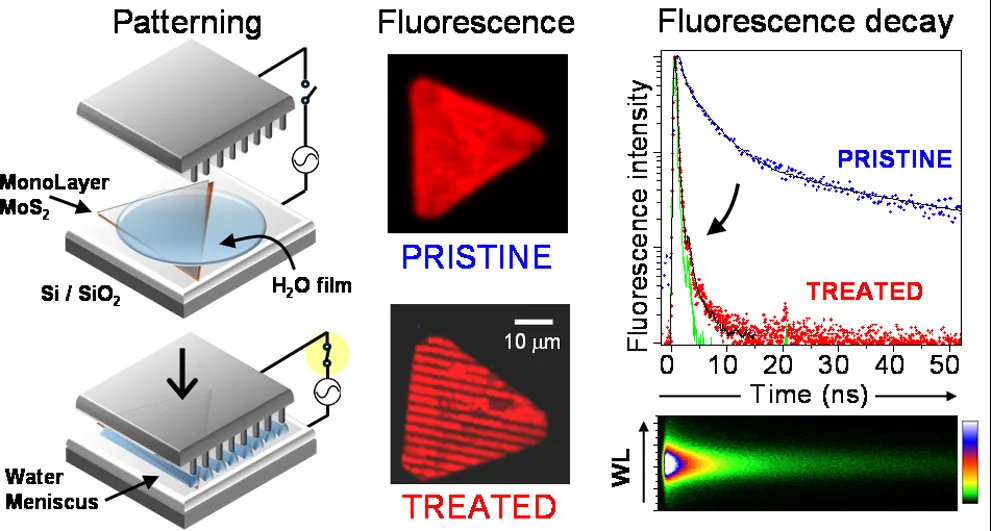

Over the past two decades, the discovery of two-dimensional (2D) materials has revolutionized many areas of materials science and condensed matter physics.^1^ Reduced dimensionality fundamentally alters the electronic structure by inducing strong quantum confinement, enhancing surface and interface effects, and giving rise to characteristic low-dimensional densities of states,^2^ thereby enabling several unique functionalities.^3–5^ However, these same features make 2D materials exceptionally sensitive to defects, so even low defect densities can dramatically alter their chemical, electronic, and optical properties.

Although defects are traditionally considered detrimental to material performance and stability, some offer unexpected opportunities that are used as an additional degree of freedom in materials engineering. In particular, atomic vacancies and heteroatoms, the most diffuse defects, introduce new functions and physical phenomena that are impossible in pristine materials; these include opportunities in ion storage,^6^ catalysis,^7^ and quantum capacitance,^8^ among others.^4,5^ Yet many defect-driven properties have been demonstrated only qualitatively, because their intensity and control are insufficient for practical applications. Therefore, defect engineering is crucial for managing and using these effects in technological and fundamental applications.

Here, we exploit this paradigm to introduce an unexplored functionality by demonstrating that a MoS_2_ monolayer can host spatially controlled and programmable long-lived charge trapping, thereby conferring electret functionality,^9^ i.e., the ability to store quasi-permanent electrostatic charge, on a MoS_2_ monolayer, a property not achieved in any actual 2D system. In addition, we fabricate a multifunctional fluorescent pattern that acts as a platform for controlling exciton emission nature and dynamics. Although charge trapping in 2D semiconductors has been observed in various 2D contexts,^10–13^ the charge densities and retention times achieved so far have been insufficient for electret applications (Table S1). Here, we addressed these limitations through fine defect engineering using electrode-free electrochemical nanolithography,^14^ a technique that allows us to generate spatially localized charged defects in 2D-MoS_2_. This approach transforms two features typically associated with reliability concerns, namely electrically induced defects^15^ and charge trapping,^10^ into a functional property in MoS_2_ monolayers crystals (2D-MoS_2_).

2D-MoS_2_ were samples grown on Si/SiO_2_ substrates by chemical vapor deposition^16^ and were characterized by optical and fluorescence microscopy (FM), atomic force microscopy (AFM), Raman, X-ray photoelectron spectroscopy (XPS), Kelvin probe force microscopy (KPFM), electric force microscopy (EFM), and time-resolved fluorescence (TRF).

To fabricate spatially defined defect domains, we used electrode-free anodic oxidation nanolithography (EFLAO),^14^ a recently developed technique for oxidative etching of 2D materials that also enables fine control over defect formation. In EFLAO, a conductive probe is brought into contact with the surface in a high-humidity environment (RH > 90%), forming a nanoscale electrochemical cell. Unlike conventional electrochemical nanolithography,^17^ EFLAO operates through capacitive coupling between the conductive probe and the sample, applying an AC bias.^14^ Originally implemented as a scanning probe technique, we adapted EFLAO for parallel processing by replacing the probe with a stamp, thereby enabling centimeter-scale patterning in a single step.^18^ Figure 1 shows the scheme of the process.

**Figure 1:**
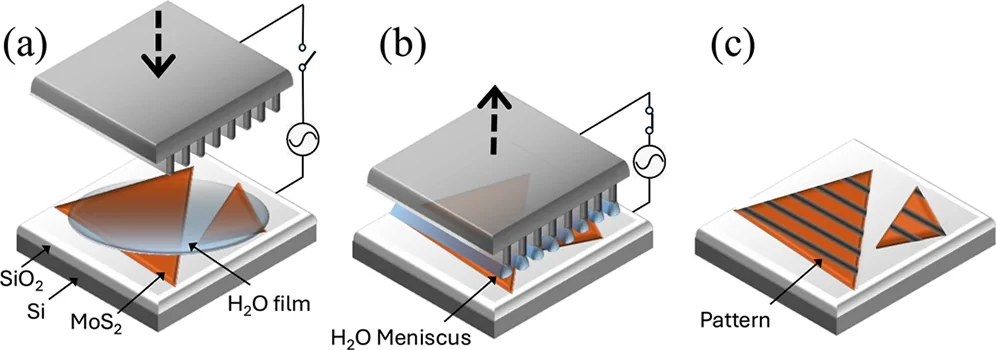
Scheme of stamp-assisted electrode-free local anodic oxidation nanolithography. (a) A thin water layer is formed in highly humid conditions (relative humidity of >95%). (b) Stamp motifs create a water meniscus through capillary force upon contact with the surface, forming an array of nanoelectrochemical cells. Applying an AC bias voltage induces electrochemical etching within these nanocells. (c) The reaction occurs exclusively beneath the stamp protrusions, resulting in the patterning of surface structures that replicate the stamp relief features.

We examined the morphological effects of EFLAO treatment on 2D-MoS_2_ using contact AFM, a setup chosen to suppress electrostatic artifacts, such as those that can occur in intermittent-contact imaging, especially when measuring crystal thickness.^19^ For treatment durations shorter than 150 min, no detectable morphological changes were observed on 2D-MoS_2_ (Figure 2a). No trace of contaminant or local reorganization was observed. At longer treatment durations, a <0.5 nm increase in local roughness was observed (Figure S2), indicating the onset of lattice deformation. For this reason, only treatments of less than 150 min were considered in subsequent analyses.

**Figure 2:**
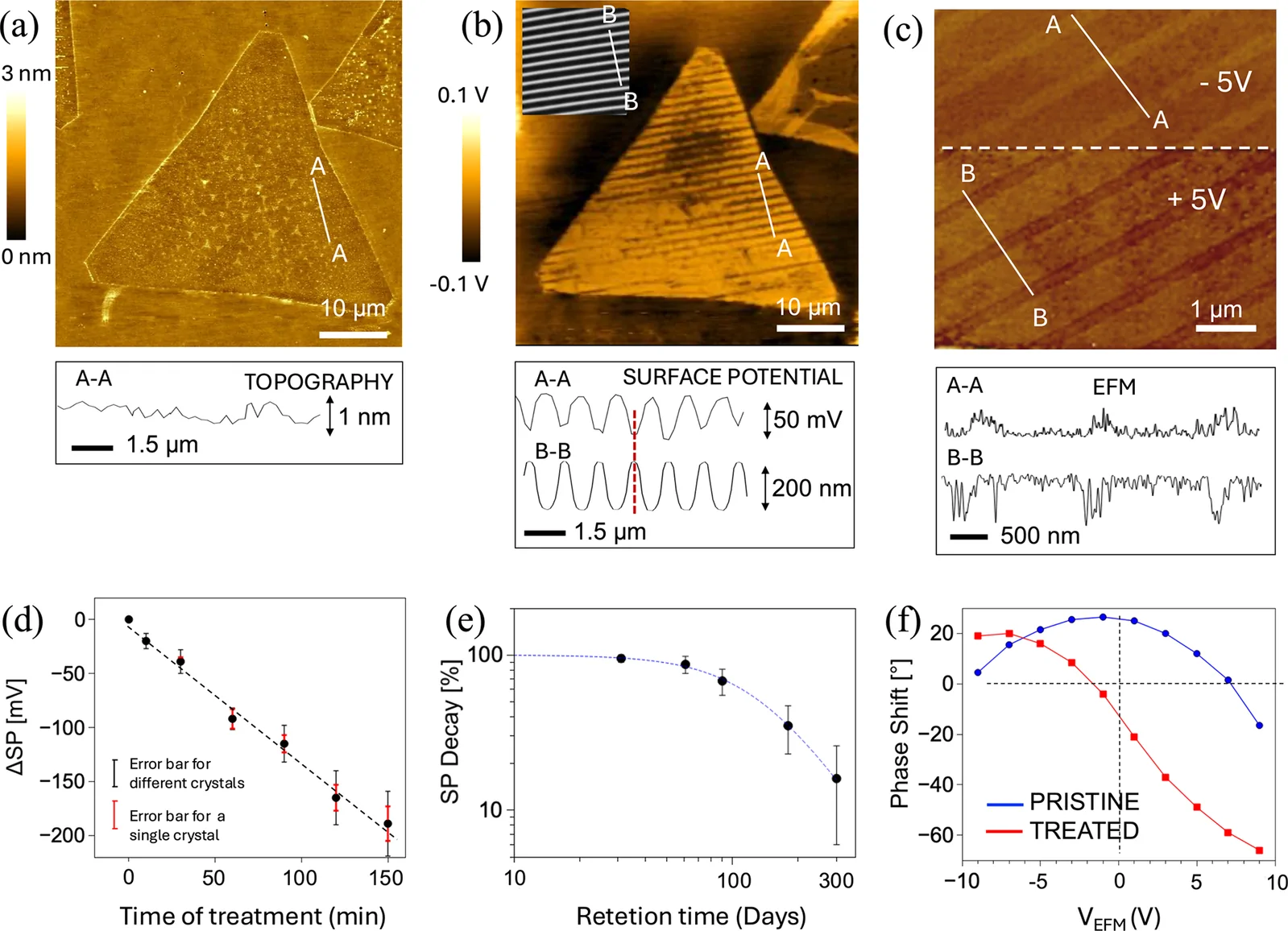
(a) Contact AFM topographic image of a MoS_2_ flake, one monolayer thick, after EFLAO treatment for 60 min, close to an untreated flake deposited on a Si/SiO_2_ substrate. (b) Corresponding surface-potential map obtained by KPFM. The inset shows a morphological image of the stamp. (c) EFM image recorded inside a treated MoS_2_ flake (phase shift). The tip–sample bias voltage is switched from −5 V to +5 V during acquisition, showing phase-contrast inversion when the bias is reversed. (d) Evolution of surface potential with respect to untreated zones upon treatment with different application times. Black bars refer to the error considering different crystals; red error bars refer to the error inside a single crystal. The dashed line is a guide for the eyes. (e) Long-term trend (expressed as percentage) of surface potential, maintaining the sample in air under ambient conditions and exposed to light. The blue curve represents the Hill-type fitting with exponent of *n* ≈ 2. f) EFM phase–bias curves for pristine 2D-MoS_2_ (blue curve) and treated (red curve) 2D-MoS_2_.

X-ray photoelectron spectroscopy (XPS) measurements show that, as is typical for CVD-grown MoS_2_, the pristine material already contains a fraction of native defects (∼5%), identified as sulfur vacancies (V_S_).^20^ Despite a small increase in the V_S_ signal (not experimentally significant, as the variation falls below the experimental detection limit of the technique), a comparison between pristine and treated 2D-MoS_2_ shows no significant changes in either binding energies or relative peak intensities (Figure S3 and Table S2), indicating that any compositional modification induced by the treatment involves less than 1% of the atomic species. Confocal Raman spectroscopy of pristine 2D-MoS_2_ reveals two characteristic first-order vibrational modes, namely, the in-plane E_2g_^1^ mode at ∼384 cm^–1^ and the out-of-plane A_1g_ mode at ∼405 cm^–1^ (Figure 3), consistent with the monolayer nature of our samples.^21^ After EFLAO treatment, the overall Raman line-shape remains essentially unchanged (Figure 3a), confirming that the crystal lattice is preserved and no significant structural degradation occurs during processing. Nonetheless, treated samples systematically display a slightly larger A_1g_–E_2g_^1^ mode separation, primarily arising from a red shift of the E_2g_^1^ peak by approximately 2.0 cm^–1^ (Figure 3b). This spectral shift is consistent with a mild, fractional desulfurization process, corresponding to an estimated average V_S_–V_S_ spacing exceeding 10 nm.^21,22^

**Figure 3:**
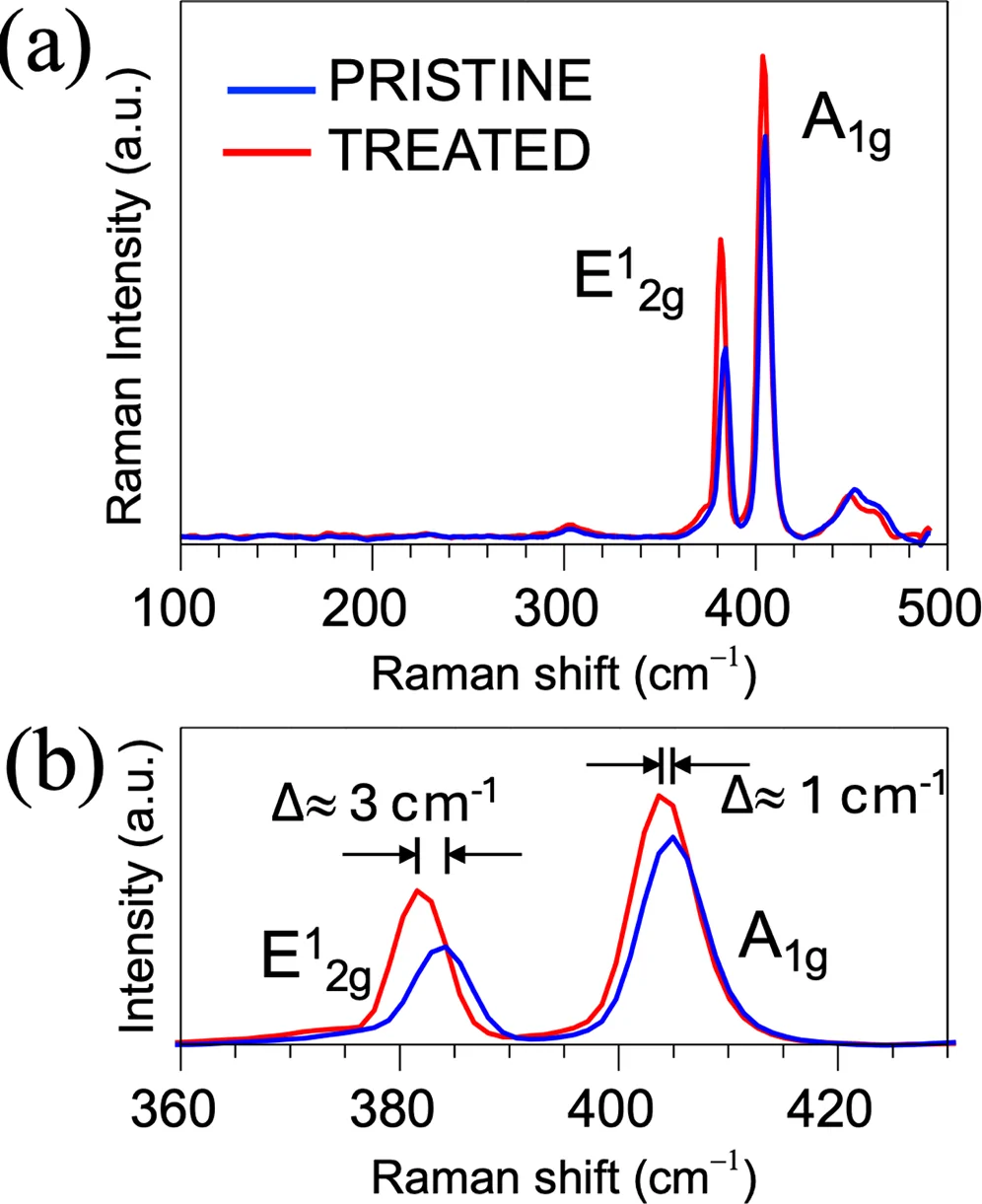
(a) Raman spectra (λ_exc_ = 532 nm) of pristine (blue) and treated (red) monolayer crystals; (b) zoom on the E_2g_^1^ and A_1g_ bands spectral region. Upon patterning, the E_2g_^1^ peak is observed to shift from <383.8 cm^–1^ to <381.7 cm^–1^ (Δλ<2.1 cm^–1^).

The morphological and spectroscopic results align with reports of moderate electrochemically assisted desulfurization of MoS_2,_ suggesting early formation of multiatomic defects that compromise MoS_2_ lattice quality during treatments longer than 150 min.^23,24^

We investigated the electronic effects of EFLAO patterning using KPFM and EFM. Unlike morphological (no)­effects, even after short treatments, the surface potential (SP) maps obtained by KPFM show a strong modulation on the 2D-MoS_2_, perfectly replicating the stamp features (Figure 2b). The treated regions form parallel stripes approximately 250 ± 50 nm wide with a 1500 nm pitch, exhibiting a more negative SP than untreated areas. Although SiO_2_ is known to exhibit electret behavior,^18^ no significant SP contrast was detected on the SiO_2_ substrate under our experimental conditions (Figure 2b), because the MoS_2_ crystals act as spacers between the stamp and the substrate.

The magnitude of the SP contrast measured within a flake (ΔSP = SP_Treated_ – SP_Untreated_) increases linearly with treatment duration, from ΔSP = −23 ± 7 mV after 10 min to ΔSP = −180 ± 30 mV after 150 min (Figure 2d). At longer durations, the onset of morphological roughening reduces measurement reproducibility. The linear increase in ΔSP aligns with a noninteracting formation of isolated atomic V_S_, resulting in a low vacancy density. Within 150 min of treatment, no signs of preferential formation of multiatomic V_S_ were observed.

The negative polarity of ΔSP indicates the accumulation of trapped negative charge (i.e., electrons) in the patterned areas. The SP contrast in correspondence with the printed lines is relatively sharp, showing strong lateral confinement and minimal diffusion of trapped charge across the monolayer. The polarity and periodicity of the potential contrast are highly reproducible across different samples, with variability within a flake below 10% (red error bars in Figure 2d). Slight differences between flakes (black error bars) arise from local variations in the stamp–surface gap, caused by multilayer regions or minor environmental fluctuations, indicating a strong sensitivity of the process to the experimental conditions and substrate quality.

Using an infinite plane electrostatic approximation,^19^ the trapped charge density was estimated to range from ∼130 ± 10 nC/cm^2^ after 10 min of treatment to −1000 ± 50 nC/cm^2^ after 150 min. These charge densities are comparable to or exceed those of many conventional electrets,^25,26^ yet they remain confined to a single monolayer. Assuming each formed defect traps one electron, the number of sulfur vacancies (N_Vs_) per surface unit increases from 8.1 × 10^11^ N_Vs_/cm^2^ after 10 min of treatment to 6.3 × 10^12^ N_Vs_/cm^2^ after 150 min, corresponding to a defect formation rate of 3.7 × 10^10^ (N_V_S__/cm^2^)/min.

Under ambient conditions (RH = 50%–75%) and light exposure, ΔSP gradually decays with aging. Within 300 days, ΔSP decreases from 100% to ∼20%, independently of its initial value (Figure 2e), probably because of V_S_ passivation by oxygen or water vapor.^27^ We attributed the residual charge observed after long aging times to traps at the MoS_2_/SiO_2_ interface,^13^ as these traps are likely less exposed to environmental effects, such as oxygen passivation, as they are partially protected by the MoS_2_ layer itself.

To phenomenologically describe the discharging process, we fitted the temporal evolution of the normalized surface potential contrast using a Hill-type function ΔSP­(*t*)/ΔSP­(*t*_0_) = [1 + (*t*/τ)^*n*^]^−1^, where ΔSP­(*t*_0_) is the initial surface potential contrast, τ is the characteristic relaxation time, and *n* is the Hill exponent, which quantifies deviations from first-order kinetics. The resulting decay is well-described by a Hill-type function (Figure 2e) with a characteristic relaxation time of τ = 134 ± 4 days and an exponent of *n* ≈ 2. We use this function as a phenomenological descriptor of the discharge kinetics. While stretched-exponential functions describe relaxation governed by a broad distribution of independent detrapping times, the Hill-type expression compactly captures the persistent retention tail observed in the vacancy-rich domains. The exponent, *n* ≈ 2, therefore, reflects a deviation from first-order kinetics, consistent with correlated relaxation within the defect-engineered MoS_2_ landscape.^28^

EFM imaging (Figure 2c) and phase-shift measurements relative to the applied bias (Figure 2f) confirmed the electrostatic origin of the patterned contrast.^29^ While pristine flakes show the typical quadratic dependence of the phase shift on the applied bias, characteristic of purely capacitive interactions (Figure 2f, blue curve), the treated regions show an almost linear dependence (Figure 2f, red curve), indicating electrostatic coupling between the probe and static surface charge. The negative phase shift further confirms the accumulation of excess negative charge in the patterned domains.^30^

Collectively, these results demonstrate the efficiency of the EFLAO treatment in precisely tailoring the local electronic properties at a submicrometric scale by creating V_S_ in the MoS_2_ lattice, which act as charge-trapping centers.

Because the optical properties of 2D-MoS_2_ are highly sensitive to the nature and concentration of defects,^31^ we conducted complementary steady-state fluorescence microimaging and time-correlated fluorescence microspectroscopy to investigate the optical effects induced by EFLAO on individual 2D-MoS_2_. All measurements were taken at the center of the crystals to avoid possible edge-related effects, which are known to influence large-area MoS_2_ monolayers.^21^

In fluorescence microspectroscopy, 2D-MoS_2_ exhibits a broad emission spectrum with a distinct band centered at ∼665 nm (1.86 eV, Figure 4c), which arises from a single emitting state associated with the A-exciton transition at the *k*-point of the Brillouin zone and with the A^–^ (negative trion), a bound state of two electrons to a hole, i.e., a negatively charged exciton.^31–33^

**Figure 4:**
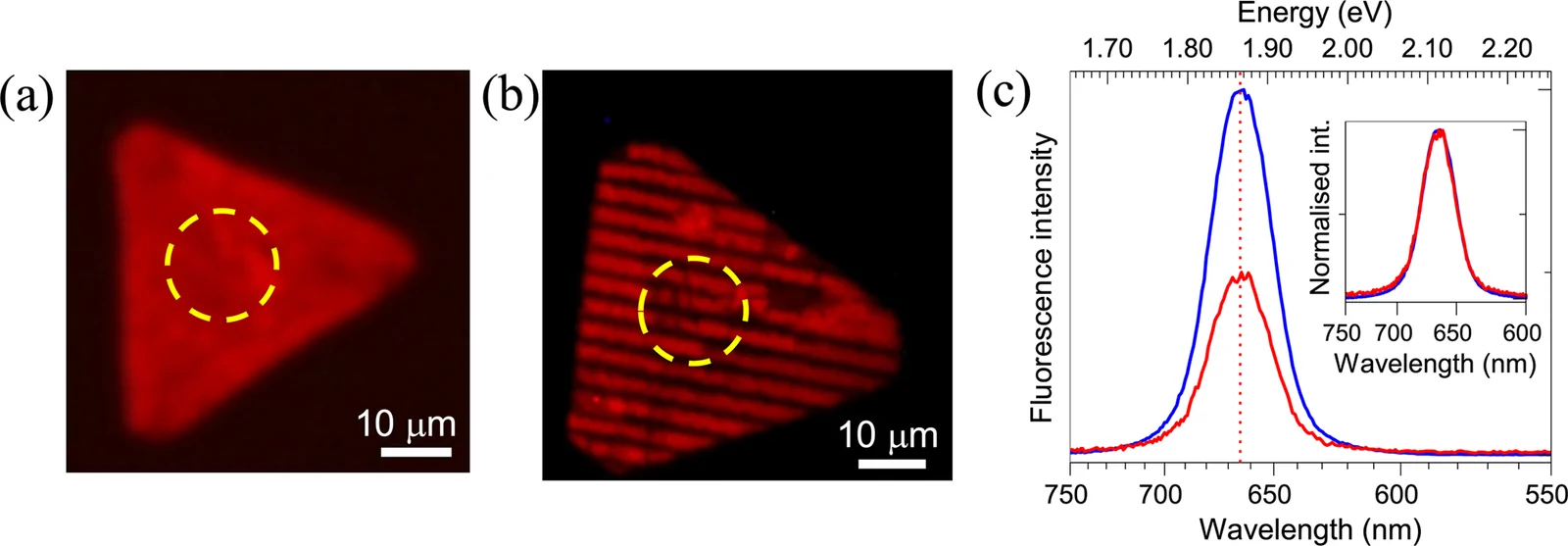
Room-temperature emission microspectroscopy. Fluorescence microimage (λ_exc_ = 535 nm, integrated detection spectral range λ_det_ > 590 nm) of (a) a pristine and (b) treated monolayer crystal of MoS_2_. The spectra were collected from the center of the crystal within a circular region (diameter ≈ 20 μm). (c) Corresponding steady-state fluorescence spectra of pristine (blue) and treated (red); (inset) relative intensity normalized plot to evidence the equivalent spectral shape.

Trions are reported to possess a large binding energy (20–30 meV), making them significantly emissive, even at room temperature.^31^ Following EFLAO treatment, fluorescence images reveal an optical pattern of parallel lines arising from fluorescence quenching (dark regions), corresponding to the treated zones identified by KPFM (Figure S4). Comparative spectral analysis (Figure 4c) shows that the overall emission intensity decreases in the treated samples as recorded at room temperature. Although the KPFM-identified treated area accounts for ∼25% of the surface, the steady-state fluorescence intensity decreases by ∼50% relative to the initial value, with no significant alteration in spectral band shape or peak position (Figure 4c, inset). This behavior indicates that the process induces strong fluorescence quenching, mainly confined to the treated regions. Additionally, the dark stripes in the fluorescence images usually appear broader than in KPFM.

We investigated exciton dynamics and recombination pathways in 2D-MoS_2_ using time-resolved fluorescence. The temporal evolution of the fluorescence spectrum of a pristine monolayer, recorded at room temperature, following a ∼450 ps (fwhm) excitation pulse (λ_exc_ = 402 nm (3.08 eV)), shows an emission band centered at ∼665 nm (∼1.86 eV). The emission of the pristine crystal decays with a relatively long time-constant (Figure 5a, observed range of 0–10 ns) while both the spectral shape and the peak position remain unchanged, even on a longer time window (Figure 5a, intensity map, observed range 0–37 ns). Fluorescence transients recorded at the emission maximum give an intensity-averaged decay time of 10–15 ns, consistent with reported values for high-quality MoS_2_ monolayer^31^ (Figure 5c). However, even in pristine crystals, we observed a multiexponential recombination behavior, indicating the presence of a pre-existing distribution of surface defects that affects the local value of MoS_2_ exciton diffusion length (see Figure S5 for decay fitting details).

**Figure 5:**
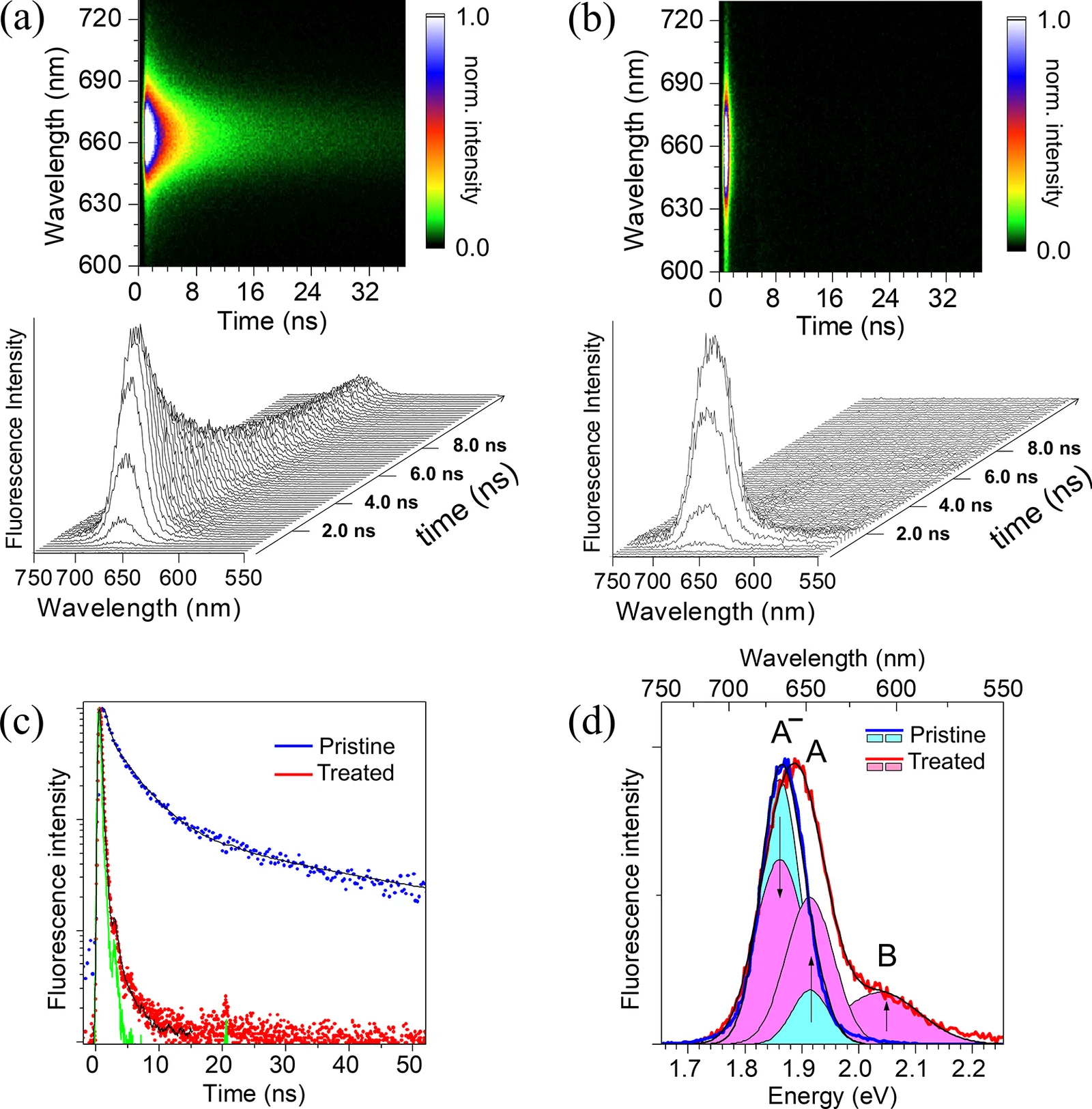
Room-temperature time-resolved fluorescence spectroscopy. (a) Sub-ns time evolution of fluorescence spectrum of *pristine* 2D-MoS_2_ under microscope (objective 100×, λ_exc_ = 402 nm (3.08 eV), *f* = 5 MHz, time separation between spectra: Δ*t* = 444 ps). False color normalized intensity map, traced up to 37 ns (Δ*t* = 74 ps). (b) Same visualization for *treated* sample. (c) Normalized microfluorescence decays both of pristine (blue) and treated (red) monolayer crystals of MoS_2_ (λ_exc_ = 402 nm, *f* = 4 MHz, λ_det_ = 665 nm, Δ*t* = 74 ps), instrumental response function (IRF, green) and relative best fit exponential convoluted curve (black line). d) *Prompt* emission spectra (time range Δ*t*: 0–1 ns) and their Gaussian deconvolution through A^–^ trion, and neutral A and B excitons. The arrows indicate the change in relative band contribution following EFLAO treatment (spectra are normalized). See text for details.

Treated crystals behave differently: their fluorescence decay becomes much faster, with characteristic times reduced to ∼180 ps (see Figure 5c and Figure S6 for details). Thanks to the high time-selectivity of correlated emission spectroscopy techniques, some spectral details, scarcely visible in a steady-state analysis (as in Figure 4c), become apparent.

Figure 5d shows the intensity-normalized time-resolved emission spectra, as integrated just within the electrect nanosecond after the excitation (Δ*t* = 0–1 ns), both for the pristine (blue curve) and treated (red curve) samples. Compared to that of the pristine sample, the spectrum of the treated one exhibits, in addition to a reduction in overall intensity: (i) a broader emission (its fwhm increases from 87.2 to 127.3 meV, Δ ≈ 40 meV), and (ii) the appearance of a shoulder at ∼605 nm (∼2.05 eV). The deconvolution of both spectral shapes through Gaussian bands corresponding to the emission of A^–^ trion (∼1.86 eV) and of A (∼1.91 eV) and B excitons (∼2.04 eV)^33,34^ is shown in the figure, in cyan for pristine and in magenta for the treated sample. It is worth noting that, while the pristine sample emission is dominated by the A^–^ trion component, with respect to the A exciton one (relative intensity ratio A^–^/A_pristine_ ≈ 5), in the treated one, this ratio decreases drastically (A^–^/A_treated_ ≈ 1.25). In fact, upon patterning, because of the strong relative intensity gain of the A exciton contribution, the overall emission spectrum of the treated sample appears, as observed, broader on its high-energy side.

The treated samples are therefore characterized, even if in an overall reduced emission intensity, by a more evident (neutral) exciton A emission together with the higher energy spin–orbit split state (B exciton), which gains relative intensity, with respect to A.

Notably, the B/A peak ratio directly reflects the effectiveness of EFLAO process, since a higher B/A value is reported to indicate the presence of a higher density of nonradiative channels in the crystal, associated with a reduction of overall emission intensity and a shortening of the A-exciton lifetime.^35^

Moreover, the EFLAO treatment seems to have the net effect of reducing the surface density of *freely moving* charges, presumably captured by trapping sites inside the printed areas, with the formation of a minor number of negatively charged A excitons, i.e., of A^–^ trions in untreated zones. These experimental findings are consistent with the introduction of a high density of V_S_ within the printed regions, acting as nonradiative exciton deactivation channels.^31^

The apparent widening of stripes in fluorescence images and the pronounced reduction in the intensity-averaged decay-time in the patterned samples can be qualitatively explained by considering the concurrent effects of defect distribution produced by the process and the natural exciton diffusion length. Because of a locally high density of (charged) defects inside the printed regions, excitons are expected to decay entirely through nonradiative pathways, creating optically nonemissive “dark zones”.

Under these conditions, the measured fluorescence is expected to originate exclusively from the unmodified regions, i.e., the “bright stripes”, which *should* retain the decay characteristics of the pristine material. Since the width of the bright stripes (∼1250 ± 50 nm) is comparable to (or slightly shorter than) the exciton diffusion length in 2D-MoS_2_ (1000–1500 nm^36^), excitons formed within the central areas of the bright stripes are expected to recombine mainly radiatively. However, because of their long diffusion length, they can reach defect-rich regions, increasing their effective nonradiative deactivation rate. Indeed, this reduces the observed fluorescence decay time. This effect increases progressively for excitons formed in proximity of the printed stripes. Excitons created near the defect-rich boundaries are almost completely quenched. As a result, dark regions appear wider in fluorescence imaging than the printed zones, and the decay curve of the treated sample becomes significantly faster than that of the pristine material. The resulting fluorescence decay reflects a continuous distribution of decay times, as excitons are generated at different distances from the defect-rich boundaries and experience progressively stronger nonradiative losses. A quantitative modeling of exciton diffusion and recombination in patterned architectures will be the subject of a dedicated publication. Notably, unlike the electric response, the suppression of optical emission in the printed area and the shortened decay time in the untreated area remain stable over time and show no evidence of aging, even after one year of shelf time. This long-term stability indicates that the quenching sites formed by EFLAO are not governed by variable charge states, supporting their assignment to V_S_. This behavior shows that nanoscale patterning can manipulate nonlocal exciton dynamics by shaping the spatial distribution of defects.

Combined SPM and spectroscopic analyses show that EFLAO treatment generates lattice defects that act as highly efficient trap centers, producing a robust and long-lived charge-trapping phenomenon in the treated MoS_2_ crystals and a permanent local quenching of fluorescence. Both experimental observations and thermodynamic considerations converge on desulfurization as the dominant reaction pathway, as V_S_ are the most readily formed defects^4^ and the process proceeds without changing the oxidation states of molybdenum or sulfur.^23^ The EFLAO process selectively forms sulfur vacancies at the stamp-defined features; these vacancies act as deep electron traps^37^ while simultaneously quenching the photoluminescence of 2D-MoS_2_.^31^ Within this framework, sulfur removal involves a coupled proton–electron transfer at surface sulfur atoms, producing gaseous H_2_S and leaving behind an atomic sulfur vacancy^23^ (Scheme S1). We hypothesize that the extreme local conditions of EFLAO activate water oxidation, thereby providing the protons and electrons necessary to sustain the desulfurization reaction and the successive electron trapping. This mechanism is consistent with reports of electrochemically assisted desulfurization in both conventional^23^ and stamp-mediated configurations^24^ and explains the V_S_ charging. However, because EFLAO cannot be treated as a conventional electrochemical system and given the extreme local conditions, we do not exclude other mechanisms.

In conclusion, we establish defect-engineered monolayer MoS_2_ as a two-dimensional electret in which precisely patterned sulfur vacancies create a stable network of electron traps. These defects retain electrostatic charge over month-long time scales under ambient conditions while concurrently reshaping exciton nature (neutral/charged) and dynamics (ns/sub-ns fluorescence lifetime). The coexistence of tunable electrostatic and optical response within a single atomic layer shows that sulfur vacancies in MoS_2_ monolayers can be harnessed as functional building blocks for surface engineering. Here, we used MoS_2_ as a model material; however, electron-trapping states associated with chalcogen vacancies are common in transition-metal dichalcogenides, including WS_2_, MoSe_2_, and WSe_2_ and other compounds, suggesting that the concept may extend to additional layered semiconductors. Future studies will systematically investigate electret formation in other materials.

## Materials and Methods

See the Supporting Information

## Supplementary Material



## Notes

Additional experimental details, materials, and methods (PDF)
