# Supplementary material for: Toward Two-Dimensional MoS2 Electrets with Modulated Fluorescence
Source: Nano Lett. 2026 Oct 16;26(43):15039–46. doi: 10.1021/acs.nanolett.6c03760 (PMC13741914; doi:10.1021/acs.nanolett.6c03760)
Supplement: Supplementary file 1 [file nl6c03760_si_001.pdf]

Toward Two-Dimensional MoS_2 Electrets with Modulated Fluorescence

Eugenio Lunedei,[1†] Andrea Liscio,[2†] Francesco Borgatti[1] Edoardo Chini[1], Pasquale D'Angelo,[3] Fiorenza Esposito,[3,4] Niccolò Borghi,[5] Matteo Mannini,[5] Denis Gentili,[1] Luca Seravalli,[3] Massimiliano Cavallini[1]**

1- Istituto per lo Studio dei Materiali Nanostrutturati (ISMN) - Consiglio Nazionale delle Ricerche (CNR) Via P. Gobetti 101, 40129 Bologna, Italy.

2- Istituto per la microelettronica e microsistemi (IMM) Consiglio Nazionale delle Ricerche (CNR), Rome unit, Via del fosso del cavaliere 100, 00133, Roma, Italy.

3- Istituto dei Materiali per l'Elettronica ed il Magnetismo (IMEM) - Consiglio Nazionale delle Ricerche (CNR) Parco Area delle Scienze, 37/A, 43124 Parma, Italy.

4- Department of Mathematical, Physical and Computer Sciences, University of Parma, Parco Area delle Scienze 7/a, 43124 Parma, Italy

5- Dipartimento di Chimica " U. Schiff' Università di Firenze, Via della Lastruccia 3-13, 50019 Sesto Fiorentino, Italy.

SUPPLEMENTARY MATERIAL

Materials and methods

Growth MoS_2 Monolayer Crystals. MoS_2 samples were prepared by CVD assisted by molybdenum liquid precursors on a SiO_2(280nm)/Si substrates at 820°C, using a spin-coated solution of $(NH_4)Mo_7O_{24}$, NaOH and Iodixanol. The substrates with the spun solution were inserted into a quartz tube and exposed to sulphur atoms carried by a nitrogen flow [1,2]. The samples consisted of triangular flakes with lateral dimensions of 10–100 µm and a thickness of 0.9 ± 0.2 nm. In some cases, additional features were observed atop the larger monolayer domains, including small crystallites, micron-scale monolayer islands 1–2 nm thick, and, rarely (<1%), multilayer pyramidal structures. Figure S1 shows typical optical micrographs recorded in bright field, dark field, and fluorescence

microscopy of the MoS_2 flakes. Fluorescence imaging reveals the quenching of PL in correspondence with crystallites and pyramidal structures (FigureFigureS1c). In Multilayer structures, the quenching is consistent with the transition in MoS_2 electronic structure from a direct bandgap in monolayer to an indirect bandgap in multilayers [3], resulting in reduced radiative recombination efficiency.

Parallel electrode-free anodic oxidation nanolithography. *2D-MoS_2* Patterning were performed using electrode-free anodic oxidation nanolithography (EFLAO)[4]. EFLAO relies on high-frequency alternating current operation (>10 kHz) and capacitive coupling, such that only a conductive stamp is required. The method was implemented in a parallel configuration[5,6]. The experiments were carried out using a custom-built setup identical to that employed for parallel electrochemical nanolithography, as described in detail elsewhere [7,8]. Relative humidity was continuously monitored using a commercial hygrometer (Thermopro). Patterning was achieved by bringing the conductive stamp into conformal contact with the sample surface and applying an alternating bias of 15 V at 50 kHz for a defined duration under high-humidity conditions (95% relative humidity). A schematic representation of the process is provided in Figure 1 of the text. The quality and morphological uniformity of the substrate were found to be critical parameters. In particular, the presence of surface outgrowths or locally thick features markedly reduced both the efficiency and reproducibility of the oxidation process.

Confocal Raman spectroscopy (µ-Raman). Micro-Raman spectra were acquired in backscattering configuration using a Renishaw InVia/1000 Raman microscope equipped with a solid-state laser operating at a wavelength of 532 nm. The excitation beam was focused onto the sample surface through a 100× objective lens, yielding a lateral spatial resolution of approximately 1 µm. All measurements were performed under ambient conditions. To minimize laser-induced thermal effects and avoid unintentional modification of the atomically thin layers, the incident laser power at the sample surface was limited to 80 µW (more detail in ref.[9]). Spectra were collected at selected points and, where needed, across extended areas of the flakes to assess spatial homogeneity. The spectral calibration of the system was routinely verified using a reference silicon substrate before data acquisition.

Stamp fabrication and characteristics. The stamp employed for the nanolithography experiments was obtained directly from the metallic reflective layer of commercially available blank compact discs, which was used without further pattern modification. This metallic layer was mechanically supported by a 3 mm-thick film of polydimethylsiloxane (PDMS; Sylgard 184, Dow Corning),

providing mechanical compliance and ensuring conformal contact with the sample surface during processing. The stamp surface exhibits a one-dimensional grating morphology originating from the optical data tracks of the compact disc. As illustrated in the inset of Figure 2b, the pattern consists of parallel linear features with a periodicity of approximately 1.5 μm. Individual lines present an apex width of about 200 nm and a groove depth of roughly 220 nm. More detail in ref.[10,11]

AFM, KPFM, EFM Measurements: Scanning probe microscopy measurements were performed using a Bruker Multimode 8 system for all atomic force microscopy (AFM), Kelvin probe force microscopy (KPFM), and Electrical force microscopy EFM characterisations. All experiments were conducted in ambient air under laboratory conditions. KPFM measurements were acquired in amplitude-modulated tapping mode using a two-pass lift configuration. During the second pass, the tip was lifted by 20–50 nm relative to the surface. Topographic and electrical images were collected simultaneously using standard acquisition parameters optimised for stable operation on the investigated samples. Post-acquisition processing was applied to compensate for piezo-scanner distortions using the Gwyddion software package. Topographic images presented in the main text were corrected using global plane levelling, whereas KPFM maps were processed using line-by-line plane levelling to improve visual clarity while preserving intrinsic contrast.[12]

Estimation of trapped charge density. The trapped charge density (σ) was estimated using a parallel-plate capacitor model[13]: $\Delta SP=\sigma h_{eff}/\varepsilon_0\varepsilon_r$. Where: ΔSP is the surface potential contrast, σ is the surface charge density, h_{eff} the effective electrostatic thickness (one monolayer ~0.85 nm) and ε_r is the relative permittivity (~5).

X-ray Photoelectron Spectroscopy. XPS measurements were performed using a non-monochromatic Al K_α X-ray radiation (VSW-TA10; Al K_α, λ = 1486.6 eV) operated at 144 W (12kV , 12 mA) and a VSW-HA100 hemispherical analyser mounting a 16-channel detector and set with a pass energy of 22eV; all the spectra were analysed using the software CasaXPS 2.3 and calibrated with respect to the adventitious carbon in the C 1*s* core level region and fixed at 248.8 eV. The spectral deconvolution analysis of Mo 3*d* signals was carried out using an asymmetric Lorentzian line shape, while for the S 2*s* and S 2*p,* a combination of a Gaussian and Lorentzian line shape was employed instead.

Steady State Fluorescence Micro spectroscopy. Fluorescence images and spectra were recorded using a modified Nikon Eclipse80i epifluorescence microscope.[14] To perform Micro spectroscopy standard trinocular turret of the microscope was customized in order to mount a home-made optical system (composed of a 50 mm focal quartz lens focused into a UV-Vis/NiR optical fibre (200-2200 nm), 550 μm core, Thorlabs) to feed the fluorescence signal into an Avantes AvaSpec-2048 CCD spectrometer (2048 pixels array, DLC UV/Vis, 200- 1100 nm range, 10 μm or 100 μm entrance

slit, software programmed. This allowed to record fluorescence images and collect the photoluminescence signal from MoS2 crystals under a 20x-100x objective. For the photoluminescence spectra, a top-mounted OSRAM Mercury Short Arc lamp (HBO) 100 W was properly energy filtered to provide a suitable excitation (λ exc=535 nm) to excite the samples across the microscope objective. The fluorescence signal collected back through the same objective was fed into the spectrometer via a dichroic mirror (570 nm) and a long-pass filter (590 nm) to suppress residual excitation stray light. Integration time ranged from 500 ms to 2000 ms. Within the same experimental session, it was possible to record a bright-field micro image, followed by a fluorescence image and the local micro-fluorescence spectrum. Fluorescence images (collected by means of a Nikon Digital Sight DS-2M camera) were recorded by using various excitation filters, dichroic mirrors and long-pass exit filters.

Time-Resolved Fluorescence micro-spectroscopy. The same microscope was used to perform pulsed micro-fluorescence experiments by exciting the sample by means of a λ=402 nm, minimum 70 ps FWHM, pulsed solid-state laser diode head (Picoquant) at a repetition rate of 4÷5 MHz.[15] The excitation light was fed via a monomode Thorlab optical fibre into the microscope and brought to the sample across an epifluorescence 100x objective. The fluorescence signal from the MoS_2 crystals was then collected back across the same objective and brought via a custom optic coupled to a Thorlabs UV/Vis fibre into a f/4, 300mm, Acton Research Spectra Pro SP-2300i triple-turret monochromator equipped with a Hamamatsu H7422-20 Peltier-cooled photosensor module, component of an inverted start-stop detection system (Picoquant, TimeHarp-100) with a 36 ps/channel time resolution and synchronously driven by an Agilent pulse generator. Transient fluorescence spectra were then reconstructed from the fluorescence transients (time range 140 ns) as collected at various wavelengths, while scanning the monochromator position from 550 to 750 nm, in 200 steps, Δλ=1 nm, integration time 3 s/point, using a 150 gr/mm 500 nm blaze diffraction grating. Fluorescence transients used for extracting the decay parameters were recorded for 600 s at a wavelength (λ_{max} = 650 nm) corresponding to the maximum intensity of the emission spectrum.

Table S1. Quantitative comparison with representative literature examples.

Material/System	Charge storage mechanism	Surface charge density	Retention time	Environment	Patterning capability	Electret behavior	Ref.
Graphene Oxide	Localized charge domains and hopping transport	not reported	several hours	Ambient air	Not proved	No	Ref. [16]
h-BN charge-trapping structures	Defect-assisted charge trapping in dielectric layers	device dependent	days-months	Vacuum/device architecture	Nanoscale writing	No	Ref. [17]
MoS_2/SiO_2 interface	Triboelectric/interfacial trapped charges acting as floating gates	not quantified	Charge decay larger than SiO_2 by 1-2 orders of magnitude; typically hours to days	Ambient AFM measurements	Nanopatterns demonstrated	No	Ref. [18]
MoS_2 FET defects	Charge trapping/detrapping at defect states (Si/O dangling bonds) at MoS_2-SiO_2 interface	not reported	Dynamic trapping/detrapping process governing hysteresis	Device operation	No spatial patterning	No	Ref. [19]
Liquid-MoS_2 tribovoltaic interfaces	Dynamic interfacial electron transfer during liquid-solid contact	not reported	Only during continuous tribovoltaic operation	Liquid-solid interface	Not reported	No	Ref. [20]
TMD charge storage devices	Floating-gate or dielectric-assisted storage	architecture dependent	days-years	Device dependent	Lithographically defined devices	No	Ref. [21]
Sulfur-vacancy MoS_2 electret	Deep trapping at engineered sulfur vacancies	~1 μC cm^{-2}	hundreds of days	Ambient air	Sub-micrometer	Yes	this work

Supplementary experimental data

Table S2: Relative percentage (%) of MoS_2 species in the pristine and EFLAO-treated samples, and semiquantitative elemental analysis extracted from spectra reported in Figure S3.

Component	***Pristine***	***EFLAO treated***
MoS_2	88.6 %	88.9 %
Defects	5.4 %	5.8 %
MoO_x	6.0 %	5.3 %
Ratio		
S/Mo	1.8	1.9

Scheme S1. Proposed chemical desulphurization triggered by water oxidation.

$$H_2O \rightarrow 2H^+ + 2e^- + \frac{1}{2}O_{2(g)}\uparrow \qquad \text{Water oxidation}$$

$$S{-}Mo{-}S^* + 2H^+ + 2e^- \rightarrow S{-}Mo{-}V_S + H_2S_{(g)}\uparrow \qquad \text{Desulfurization } (V_S = \text{sulfur vacancy})$$

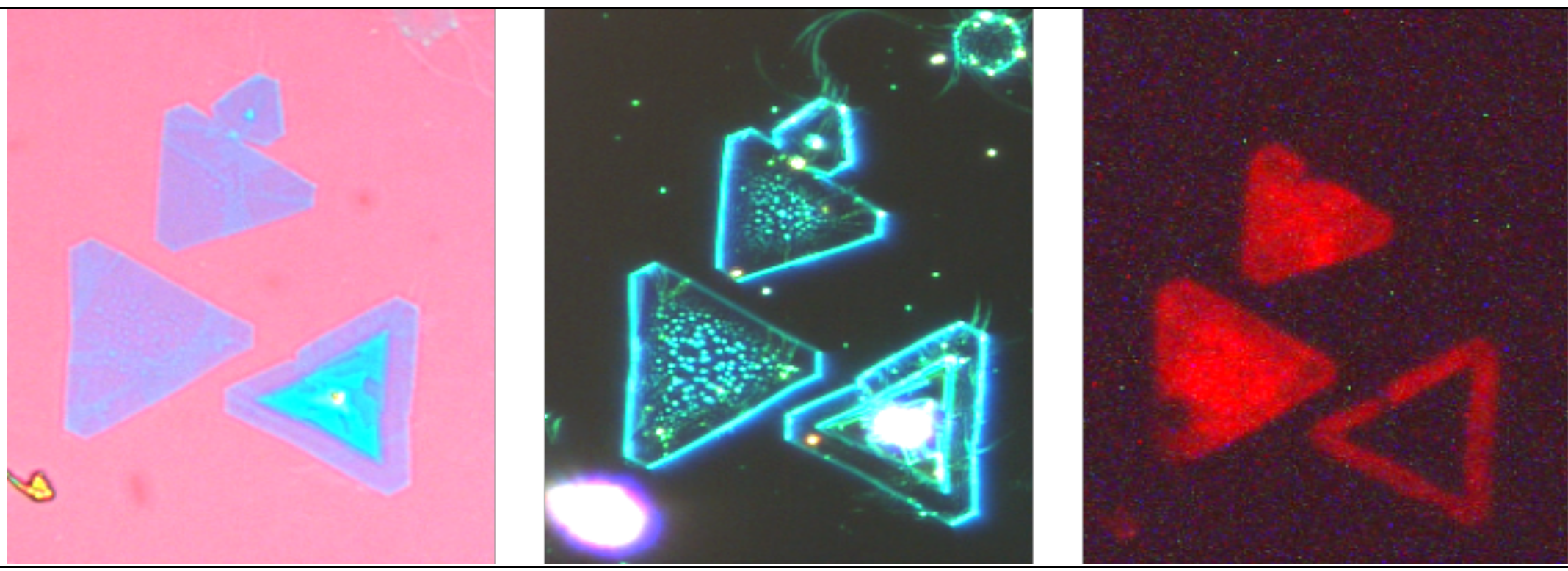

Figure S1. Optical characterization of MoS_2 monolayer crystals. (a) Optical image recorded in the bright field of MoS_2 flakes deposited on thermal SiO_2. The image shows a particular zone containing a flake with multilayers in the center and small triangular crystallites on top of the flakes (poorly visible in bright field OM). (b) Corresponding optical image recorded in dark-field. The image highlights the multi-layered structure and clearly evidence small triangular layers. (c) Corresponding fluorescence image showing the fluorescence of flake and the quenching effects of multiLayer structures and flakes.

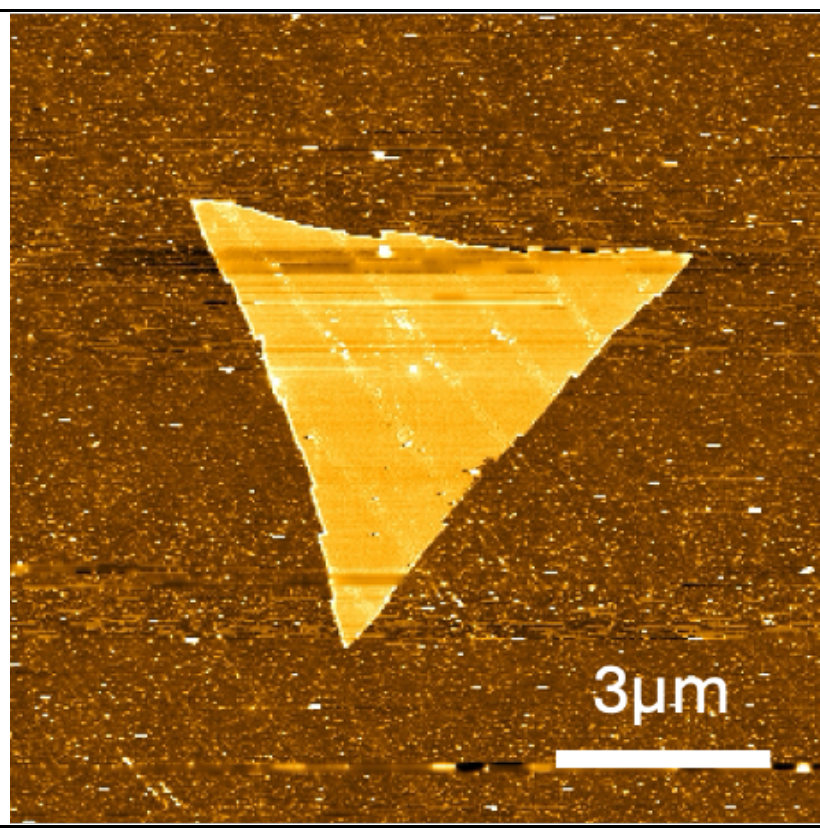

Figure S2. Topography of 2DMoS2 crystals treated for 180 mins

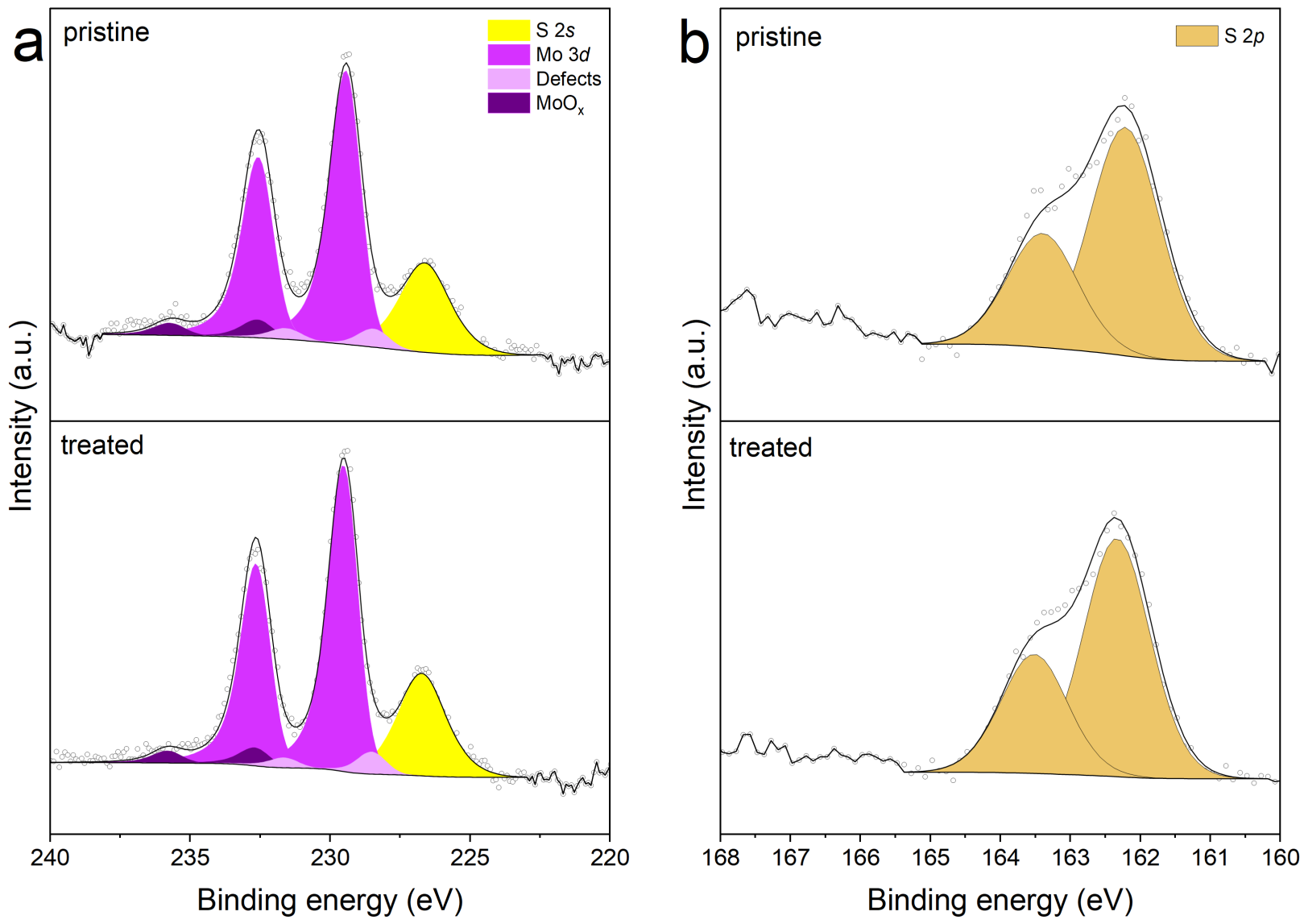

Figure S3. (**A**) XPS spectra of the Mo 3*d*, S 2*s* (**A**) and S *2p* (**B**) core level region prior and after the EFLAO treatment.

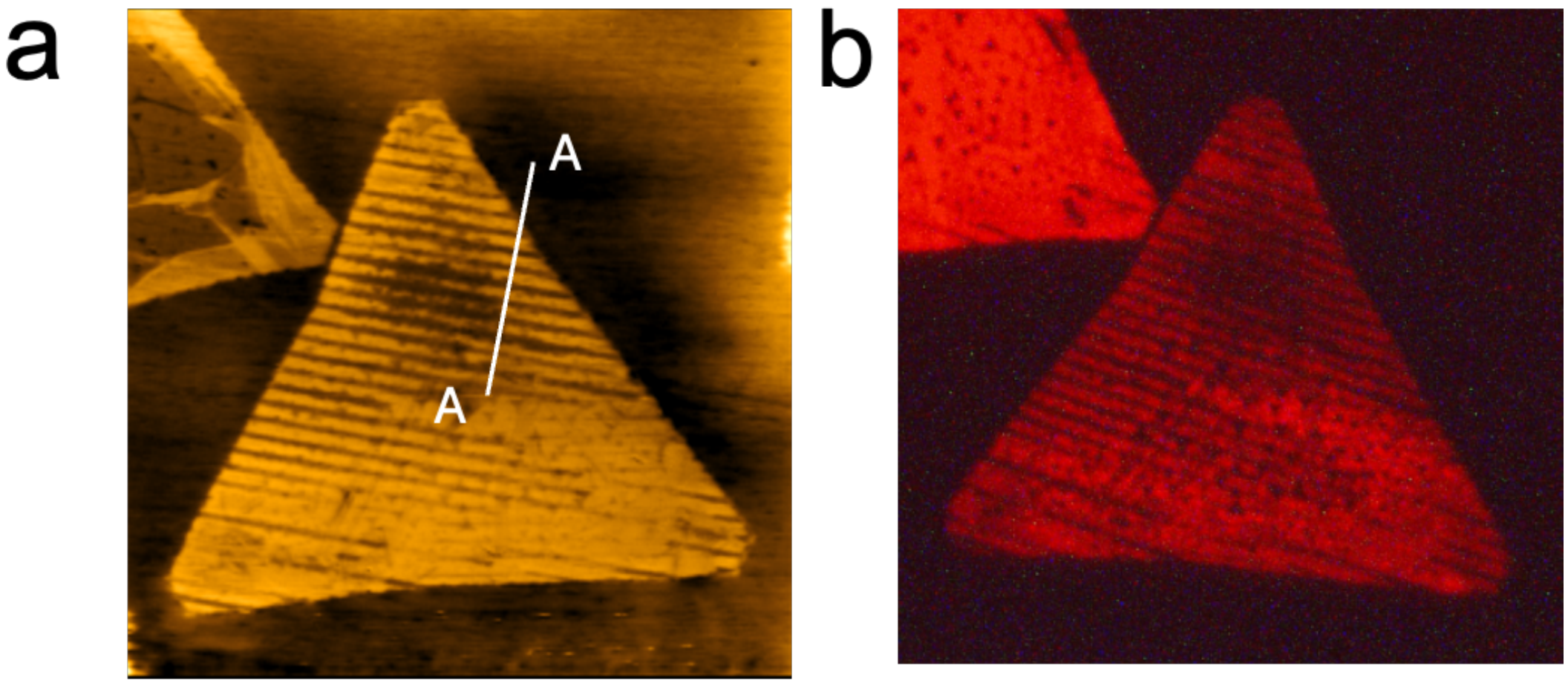

Figure S4. Direct comparison from surface potential images (a) and fluorescence microscopy image (b).

.

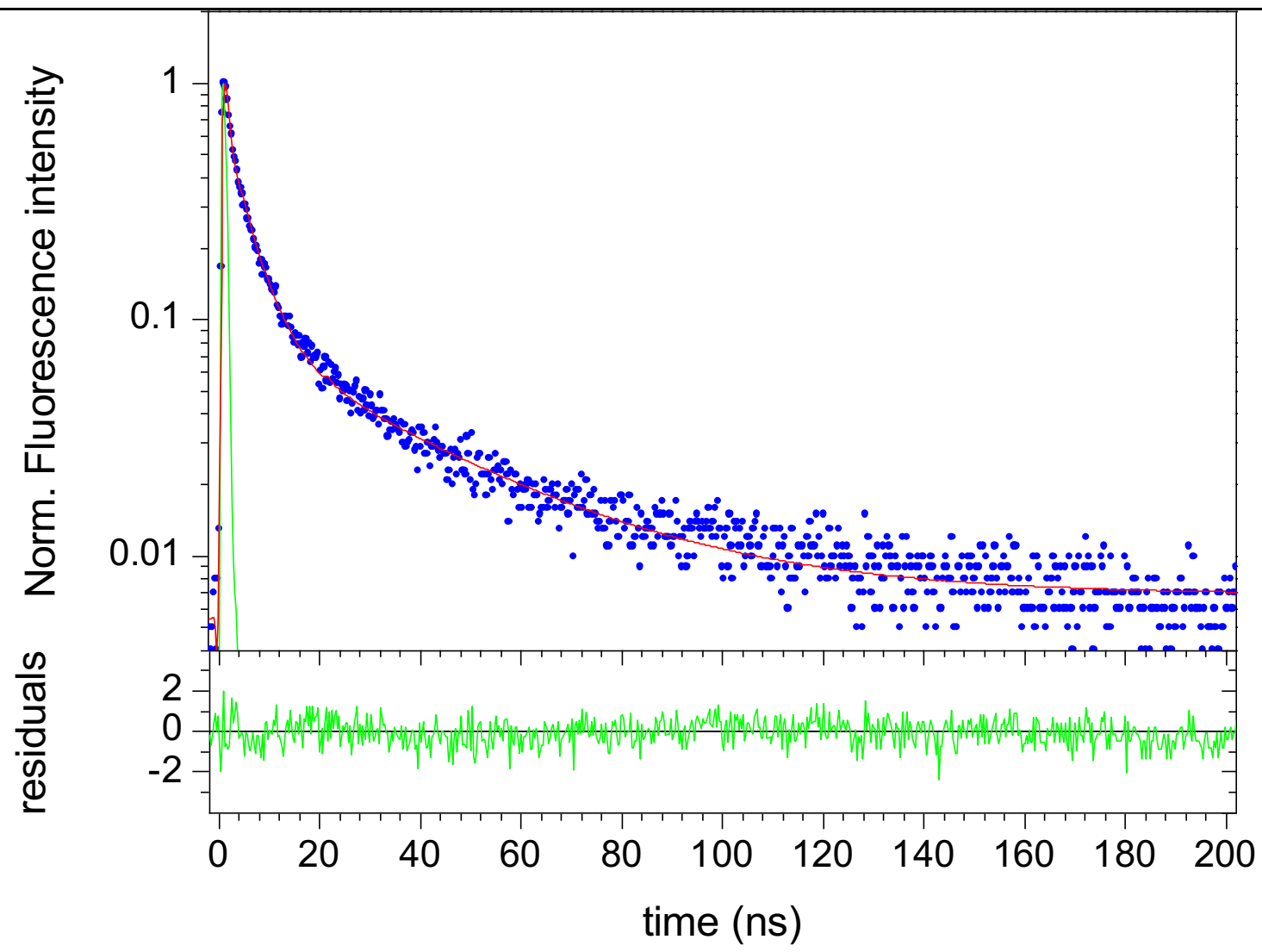

Figure S5- Pristine MoS_2 monolayer crystal, fluorescence decay fitting parameters.

Fitting parameters:

A_1	=	633.4	Cnts
t_1	=	4.308	ns
A_2	=	2197.3	Cnts
t_2	=	0.448	ns
A_3	=	85	Cnts
t_3	=	32.38	ns
Bkg.Dec	=	3.7	Cnts
Bkg.IRF	=	-0.5	Cnts
ShiftIRF	=	-86.635	ns

Average Lifetime:

$\tau_{av\text{-}1}$ = 15.669 ns (intensity weighted)
$\tau_{av\text{-}2}$ = 2.217 ns (amplitude weighted)

Fractional Intensities of the Positive Decay Components:

t_1	(4.308 ns):	42.21%
t_2	(0.448 ns):	15.23%
t_3	(32.380 ns):	42.56%

Fractional Amplitudes of the Positive Decay Components:

t_1	(4.308 ns):	21.72%
t_2	(0.448 ns):	75.36%
t_3	(32.380 ns):	2.91%

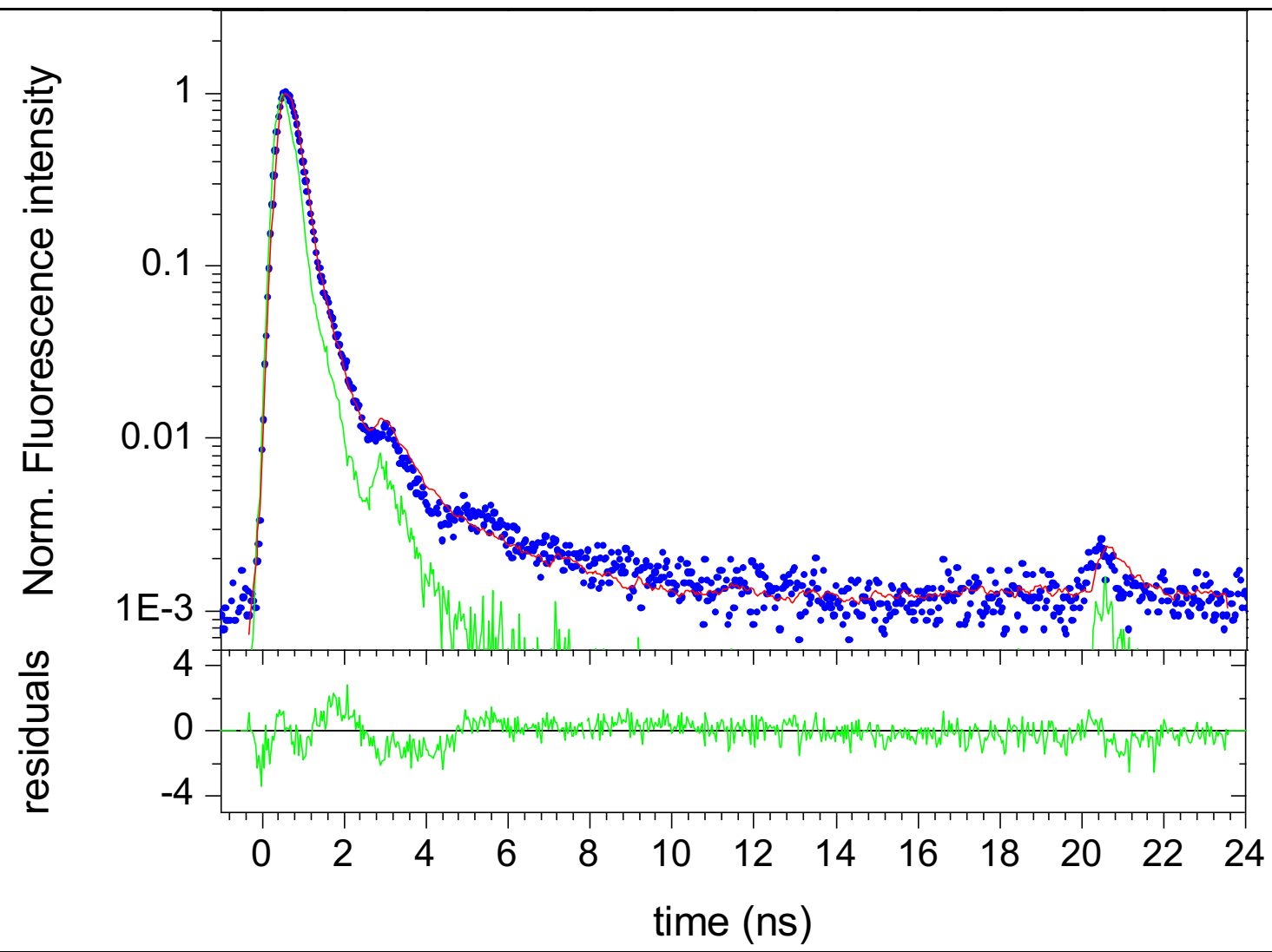

Figure S6 – Treated MoS_2 monolayer crystal, fluorescence decay fitting parameters

Fitting parameters:

A_1	=	120017.6	Cnts
t_1	=	0.129	ns
A_2	=	275.6	Cnts
t_2	=	1.792	ns
Bkg.Dec	=	9	Cnts
Bkg.IRF	=	-5.3	Cnts
ShiftIRF	=	-0.043	ns

Average Lifetime:

$\tau_{av.1}$ =0.181	ns	(intensity weighted)
$\tau_{av.2}$ =0.133	ns	(amplitude weighted)

Fractional Intensities of the Positive Decay Components:

t_1	(0.129 ns):	96.92%
t_2	(1.792 ns):	3.08%

Fractional Amplitudes of the Positive Decay Components:

t_1	(0.129 ns):	99.77%
t_2	(1.792 ns):	0.23%

Supporting Information References

(1) Esposito, F.; Bosi, M.; Attolini, G.; Rossi, F.; Panasci, S. E.; Fiorenza, P.; Giannazzo, F.; Fabbri, F.; Seravalli, L. Role of density gradients in the growth dynamics of 2-dimensional MoS using liquid phase molybdenum precursor in chemical vapor deposition. *Appl Surf Sci* **2023**, *639*.

(2) Seravalli, L.; Esposito, F.; Bosi, M.; Aversa, L.; Trevisi, G.; Verucchi, R.; Lazzarini, L.; Rossi, F.; Fabbri, F. Built-in tensile strain dependence on the lateral size of monolayer MoS2 synthesized by liquid precursor chemical vapor deposition. *Nanoscale* **2023**, *15*, 14669-14678.

(3) Wang, T.; Zhang, Y.; Liu, Y.; Li, J.; Liu, D.; Luo, J.; Ge, K. Layer-Number-Dependent Exciton Recombination Behaviors of MoS2 Determined by Fluorescence-Lifetime Imaging Microscopy. *The Journal of Physical Chemistry C* **2018**, *122*, 18651-18658.

(4) Li, H. Y.; Ying, Z.; Lyu, B. S.; Deng, A. L.; Wang, L. L.; Taniguchi, T.; Watanabe, K.; Shi, Z. W. Electrode-Free Anodic Oxidation Nanolithography of Low-Dimensional Materials. *Nano Lett* **2018**, *18*, 8011-8015.

(5) Gentili, D.; Chini, E.; Cavallini, M. Generation and Tuning of Semiconductor Electronic and Functional Properties through Electrochemical Patterning. *Acc. Mater. Res.* **2025**, *6*, 1094-1104.

(6) Simeone, F. C.; Albonetti, C.; Cavallini, M. Progress in Micro- and Nanopatterning via Electrochemical Lithography. *J. Phys. Chem. C* **2009**, *113*, 18987-18994.

(7) Chini, E.; Gentili, D.; Liscio, A.; Cavallini, M. SiO2 Electret Formation via Stamp-Assisted Capacitive Coupling: A Chemophysical Surface Functionalisation. *Inorganics* **2026**, *14*, 21.

(8) Cavallini, M.; Simeone, F. C.; Borgatti, F.; Albonetti, C.; Morandi, V.; Sangregorio, C.; Innocenti, C.; Pineider, F.; Annese, E.; Panaccione, G.; Pasquali, L. Additive nanoscale embedding of functional nanoparticles on silicon surface. *Nanoscale* **2010**, *2*, 2069-2072.

(9) Juráková, J.; Midlíková, J. D.; Hrubý, J.; Kliuikov, A.; Santana, V. T.; Pavlik, J.; Moncoľ, J.; Čižmár, E.; Orlita, M.; Mohelský, I. Pentacoordinate cobalt (II) single ion magnets with pendant alkyl chains: shall we go for chloride or bromide? *Inorg. Chem. Front.* **2022**, *9*, 1179-1194.

(10) Di Maria, F.; Olivelli, P.; Gazzano, M.; Zanelli, A.; Biasiucci, M.; Gigli, G.; Gentili, D.; D'Angelo, P.; Cavallini, M.; Barbarella, G. A Successful Chemical Strategy To Induce Oligothiophene Self-Assembly into Fibers with Tunable Shape and Function. *J. Am. Chem. Soc.* **2011**, *133*, 8654-8661.

(11) Melucci, M.; Zambianchi, M.; Favaretto, L.; Palermo, V.; Treossi, E.; Montalti, M.; Bonacchi, S.; Cavallini, M. Multicolor, large-area fluorescence sensing through oligothiophene-self-assembled monolayers. *Chem. Commun.* **2011**, *47*, 1689-1691

(12) Corradini, V.; Menozzi, C.; Cavallini, M.; Biscarini, F.; Betti, M. G.; Mariani, C. Growth morphology and electronic structure of 2D ordered pentacene on the Au(110)-(1 x 2) surface. *Surf. Sci.* **2003**, *532*, 249-254.

(13) García, R.; Pérez, R. Dynamic atomic force microscopy methods. *Surf. Sci. Rep.* **2002**, *47*, 197-301.

(14) Melucci, M.; Barbarella, G.; Zambianchi, M.; Benzi, M.; Biscarini, F.; Cavallini, M.; Bongini, A.; Fabbroni, S.; Mazzeo, M.; Anni, M.; Gigli, G. Poly(alpha-vinyl-omega-alkyloligothiophene) side-chain polymers. Synthesis, fluorescence, and morphology. *Macromolecules* **2004**, *37*, 5692-5702.

(15) Zangoli, M.; Mazzaro, R.; Lunedei, E.; Fabiano, E.; Manet, I.; Candini, A.; Kovtun, A.; Goudjil, M.; Zanelli, A.; Rozen, S.; Gazzano, M.; Baroncini, M.; Di Maria, F. Pseudomorphic Transformation in Nanostructured Thiophene-Based Materials. *ACS Nano* **2025**, *19*, 2245-2260.

(16) Palacios-Lidón, E.; Colchero, J.; Ortuno, M.; Colom, E.; Benito, A. M.; Maser, W. K.; Somoza, A. s. M. Nanoscale Charge Density and Dynamics in Graphene Oxide. *ACS Mater. Lett.* **2021**, *3*, 1826-1831.

(17) Prasad, M. K.; Al-Ani, O. A.; Goss, J. P.; Mar, J. D. Charge transfer due to defects in hexagonal boron nitride/graphene heterostructures: An ab initio study. *Phys. Rev. Mater.* **2023**, *7*, 094003.

(18) Xu, R.; Ye, S. L.; Xu, K. Q.; Lei, L.; Hussain, S.; Zheng, Z. Y.; Pang, F.; Xing, S. Y.; Liu, X. M.; Ji, W.; Cheng, Z. H. Nanoscale charge transfer and diffusion at the MoS /SiO interface by atomic force microscopy: contact injection versus triboelectrification. *Nanotechnology* **2018**, *29*, 355701.

(19) Ma, X.; Liu, Y.-Y.; Zeng, L.; Chen, J.; Wang, R.; Wang, L.-W.; Wu, Y.; Jiang, X. Defects Induced Charge Trapping/Detrapping and Hysteresis Phenomenon in MoS2 Field-Effect Transistors: Mechanism Revealed by Anharmonic Marcus Charge Transfer Theory. *ACS App. Mater. Interf.* **2022**, *14*, 2185-2193.

(20) Xie, Y.; You, Q.; Bo, W.; Jiang, T.; Zheng, M.; Wang, P.; Liang, X.; Wang, Z. L. Tribovoltaic Effect at Liquid–MoS2 Interfaces and Spectral Analysis of Interfacial Charge Transfer. *Adv. Mater.* **2025**, *37*, 2506186.

(21) Shen, Y.; Zhu, K.; Xiao, Y.; Waldhör, D.; Basher, A. H.; Knobloch, T.; Pazos, S.; Liang, X.; Zheng, W.; Yuan, Y.; Roldan, J. B.; Schwingenschlögl, U.; Tian, H.; Wu, H.; Schranghamer, T. F.; Trainor, N.; Redwing, J. M.; Das, S.; Grasser, T.; Lanza, M. Two-dimensional-materials-based transistors using hexagonal boron nitride dielectrics and metal gate electrodes with high cohesive energy. *Nat Electr.* **2024**, *7*, 856-867.
